# Role of *Litsea cubeba* Essential Oil in Agricultural Products Safety: Antioxidant and Antimicrobial Applications

**DOI:** 10.3390/plants11111504

**Published:** 2022-06-03

**Authors:** Petra Borotová, Lucia Galovičová, Nenad L. Vukovic, Milena Vukic, Simona Kunová, Paweł Hanus, Przemysław Łukasz Kowalczewski, Ladislav Bakay, Miroslava Kačániová

**Affiliations:** 1 AgroBioTech Research Centre, Slovak University of Agriculture, Tr. A. Hlinku 2, 94976 Nitra, Slovakia; 2 Institute of Applied Biology, Faculty of Biotechnology and Food Sciences, Slovak University of Agriculture in Nitra, Tr. A. Hlinku 2, 94976 Nitra, Slovakia; 3 Institute of Horticulture, Faculty of Horticulture and Landscape Engineering, Slovak University of Agriculture, Tr. A. Hlinku 2, 94976 Nitra, Slovakia; l.galovicova95@gmail.com; 4 Department of Chemistry, Faculty of Science, University of Kragujevac, 34000 Kragujevac, Serbia; nvchem@yahoo.com (N.L.V.); milena.vukic@pmf.kg.ac.rs (M.V.); 5 Institute of Food Sciences, Faculty of Biotechnology and Food Sciences, Slovak University of Agriculture, Tr. A. Hlinku 2, 94976 Nitra, Slovakia; simona.kunova@uniag.sk; 6 Department of Food Technology and Human Nutrition, Institute of Food and Nutrition Technology, University of Rzeszow, 35-959 Rzeszow, Poland; hanuspawel@gmail.com; 7 Department of Food Technology of Plant Origin, Poznań University of Life Sciences, 31 Wojska Polskiego St., 60-624 Poznan, Poland; przemyslaw.kowalczewski@up.poznan.pl; 8 Department of Planting Design and Maintenance, Slovak University of Agriculture, Tr. A. Hlinku 2, 94976 Nitra, Slovakia; ladislav.bakay@uniag.sk; 9 Department of Bioenergy, Food Technology and Microbiology, Institute of Food Technology and Nutrition, University of Rzeszow, 4 Zelwerowicza, 35601 Rzeszow, Poland

**Keywords:** *Litsea cubeba*, antioxidant, antimicrobial, anti-insect, gas phase, food preservation

## Abstract

The essential oil from *Litsea cubeba* (LCEO) has good antioxidant, antimicrobial, anti-insect properties, which gives it the potential for use as a natural additive to food resources and food products in order to prevent spoilage and extend shelf life. In this study the biological activity related to food preservation was observed. The main volatile organic compounds were geranial (39.4%), neral (29.5%), and limonene (14.3%). Antioxidant activity was 30.9%, which was equal to 167.94 µg of Trolox per mL of sample. Antimicrobial activity showed the strongest inhibition against *Serratia marcescens* by disk diffusion method and minimum inhibitory concentrations MIC 50 and MIC 90 were the lowest for *Micrococcus luteus* with values 1.46 and 3.52 µL/mL, respectively. Antimicrobial activity of the LCEO vapor phase showed strong inhibition of microorganisms on apples, pears, potatoes, and kohlrabies. Over 50% of gram-positive and gram-negative bacteria and yeasts were inhibited by a concentration of 500 µL/mL. The inhibition of microorganisms was concentration dependent. Anti-insect activity was also strong, with 100% lethality of *Pyrrhocoris* *apterus* at a concentration of 25%. These results suggest that LCEO could be potentially used as a food preservative.

## 1. Introduction

Nowadays, the desire to observe and use natural substances instead of artificial ones for various purposes has been growing [1,2,3,4]. Essential oils are liquids obtained from medicinal and aromatic plants full of volatile organic compounds, especially of the Lamiaceae family [5], full of monoterpenes, sesquiterpenes, and their derivates [6]. These bioactive substances have good potential due to their antibacterial, antifungal, bactericidal, fungicidal, and antioxidant properties, which are related to the evaluation of quality of food resources and food products [7]. Microbiological quality is connected to the microbiome of the plant resources, which can be obtained as contamination from soil or water [8]. Pathogenic bacteria can cause food spoilage, which leads to a deterioration of food quality and an increase in health risks [9]. Essential oils can serve as natural antimicrobials that can inhibit microbial growth and thus improve the shelf life of plants or their products [10]. Moreover, they can inhibit foodborne pathogenic bacteria like *E. coli*, *Salmonella* species, and *L. monocytogenes*, which can cause acute gastrointestinal diseases [11,12].

*Litsea cubeba* (Lour.) Pers., the aromatic litsea, is an evergreen tree or shrub which belongs to the Lauraceae family, which is native to subtropical and tropical regions of Asia. This plant has been used in traditional medicine for many years in order to cure various diseases [13]. The essential oil from *Litsea cubeba* (LCEO) has a light-yellow color and an intense lemonish, fresh aroma. It has found an application in cosmetics [14] and has wide pharmacological utilization [15]. The chemical composition of LCEO greatly contributes to its biological properties, which can vary depending on the plant organ (e.g., fruit, leaf, flower, and root) from which the oil is extracted, as well as on harvesting time and distribution area. The main components of LCEO from fruit are citral and d-limonene [16,17]. 

Due to its bioactive components, LCEO can be used in preservation of food resources and food products. Microbial stability was improved by LCEO use on pear, and lead to an increase of shelf life [18]. The antimicrobial activity of LCEO was observed in food systems against microorganisms, which causes spoilage of food products. *Vibrio parahaemolyticus* was inhibited in oysters, *Listeria monocytogenes* was inhibited in tofu, *Lactobacillus plantarum* was inhibited in orange–milk beverage and fungi, and *Wickerhamomyces anomalus* was inhibited in soy sauce [19]. LCEO added to vegetable juices can also inhibit enterohemorrhagic *Esherichia coli*, a foodborne pathogen responsible for gastrointestinal diseases. LCEO was active against pathogenic fungi that cause spoilage of fruits and vegetables [20]. Moreover, LCEO incorporated into to package films was able to inhibit bacteria *Escherichia coli* and *Staphylococcus aureus* and fungi *Saccharomyces cerevisiae* and *Aspergillus niger* on strawberries [21]. 

Another aspect of food safety is the presence of various pests on fruits and vegetables or other stored products. LCEO has a repellent quality as well. Various fumigant activities were observed against *Lasioderma serricorne* and *Liposcelis bostrychophila,* which are responsible for the destruction of stored products [22]. LCEO is also active against *Sitophilus zeamais,* the pest on cereal that can affect the quality of grains [23]. Moreover, LCEO has an acaricidal effect against dust mites *Dermatophagoides farinae* and *D. pteronyssinus,* and stored food mite *Tyrophagus putrescentiae* [24]. 

The aim of the study was to observe the properties of LCEO related to food preservation and sustaining microbiological quality. The chemical composition was evaluated, and the antioxidant activity was determined. Antimicrobial activity of LCEO was observed both in vitro and directly on the various fruits and vegetables. Moreover, anti-insect activity was confirmed against insect *Pyrrhocoris apterus*. This is the first study where the vapor phase of LCEO was examined against the growth of the bacteria and yeasts on the food models. Moreover, the anti-insect activity of LCEO has never been tested against insects from the Pyrrhocoridae family.

## 2. Results

### 2.1. Analysis of Chemical Composition

The LCEO consisted of 27 volatile organic molecules (Table 1, 10 trace components with percentage below 0.1% are not listed). The main components of the LCEO were geranial (39.4%) and neral (29.5%), which are isomers commonly named together as citral. The next component was d-limonene (14.3%).

### 2.2. Antioxidant Activity

The antioxidant capacity of LCEO measured by quenching of 2,2-diphenyl-1-picrylhydrazyl (DPPH) radical was determined at 30.9% which was equivalent to 167.94 µg of Trolox to mL of sample (TEAC).

### 2.3. Antimicrobial Activity In Vitro

The inhibition activity of LCEO against chosen microorganism is expressed in Table 2 as the zone of inhibition and as Minimum Inhibitory Concentration (MIC). The strong activity was determined against both tested G^−^ bacteria (*A. chroococcum* and *S. marcescens*) and against G^+^ bacteria *P. megaterium*. The inhibition zone of *A. chroococcum* was 11.33 mm with MIC 50 set at 6.35 μL/mL and MIC 90 set at 8.42 μL/mL. *S. marcescens* reached the inhibition zone 14.33 mm with MIC 50 concentration 2.36 μL/mL and MIC 90 concentration 4.28 μL/mL. *P. megaterium* had an inhibition zone of 11.33 mm and MIC 50 and MIC 90 values were determined to be 3.46 and 26.54 μL/mL, respectively. The antimicrobial activity against G^+^ bacteria *M. luteus* and both tested yeasts (*C. glabrata* and *C. tropicalis)* was determined to be moderate by disk diffusion method, while *M. luteus* showed the lowest MIC 50 and MIC 90 values, determined to be 1.46 and 3.52 µL/mL, respectively. 

### 2.4. Antimicrobial Activity In Situ

Firstly, all the obtained data of antimicrobial activity were analyzed to check whether they were parametrical. The returned values ranged in the case of antibacterial activity from 0.14 to 0.98 for apple; 0.07 to 0.72 for pear; 0.17 to 0.91 for potato and 0.47 to 0.99 for kohlrabi. In the case of antifungal variability, the values ranged from 0.12 up to 0.92 for apple; from 0.14 up to 0.81 for pear; from 0.24 up to 0.93 for potato and from 0.06 up to 0.96 for kohlrabi. Subsequently, ANOVAs were calculated to interpret the obtained data further. 

#### 2.4.1. Apple

The antibacterial activity of LCEO on an apple (Table 3) at concentration 500 μL/L LCEO was the strongest against *S. marcescens* with 76.16% inhibition. Overall, the concentrations 65.2, 125, and 250 μL/L had the highest inhibition against *A. chroococcum* with inhibition 26.63, 33.23, and 48.71% respectively. The lowest inhibition activity on the apple was visible at a concentration of 62.2 μL/L on *M. luteus* with only 6.39% inhibition. Strong inhibition above 50% was visible only at a concentration of 500 μL/L against all microorganisms.

The higher yeast growth inhibition on an apple (Table 4) was evaluated against *C. glabrata* with 4.38, 11.43, 26.42, and 53.91% at concentrations 65.2, 125, 250, and 500 μL/L, respectively. The inhibition of *C. glabrata* was stronger compared to *C. tropicalis.* The strong activity above 50% was visible only on *C. glabrata* at a concentration of 500 μL/L.

#### 2.4.2. Pear

The highest bacterial inhibition on a pear (Table 5) was observed at a concentration of 500 μL/L against *S. marcescens* 76.16% and against *P. megaterium* 76.01%. Overall, *M. luteus* seemed to be inhibited the least by all concentrations where 500 μL/L inhibited the bacteria by 45.51%. Strong activity above 50% was visible in *A. chroococcum, P. megaterium,* and *S. marcescens* at a concentration of 500 μL/L.

The antimicrobial activity of LCEO against yeasts on the pear (Table 6) was weaker against *C. glabrata* at every concentration in the range 2.46% to 22.47%. For *C. tropicalis* the inhibition activity ranged with increasing concentration from 12.62% to 87.02%. The strong activity above 50% was visible only in *C. tropicalis* in concentration 500 μL/L.

#### 2.4.3. Potato

The bacterial growth inhibition of LCEO on a potato (Table 7) showed the highest inhibition activity in *M. luteus* at all concentrations in the range from 12.68% to 85.32% with increasing LCEO content. Strong activity above 50% was visible at all microorganisms in concentrations of 500 μL/L.

The activity of LCEO against yeast on the potato (Table 8) was very similar for both tested yeasts. The highest inhibition was visible at a concentration of 500 μL/L in *C. tropicalis* with 68.45% inhibition. Both *Candida* species showed strong inhibitions above 50% with the concentration of 500 μL/L.

#### 2.4.4. Kohlrabi

The activity of LCEO against bacteria on Kohlrabi (Table 9) showed the strongest inhibition against *P. megaterium*. At concentrations 250 and 500 μL/L the inhibition was highest with percentages 47.20% and 87.70%, respectively, while at a concentration of 62.5 μL/L the inhibition was the lowest with a percentage of 5.47%. The strong activity above 50% was visible for all microorganisms at a concentration of 500 μL/L.

The inhibition activity of LCEO against yeasts on kohlrabi (Table 10) was slightly higher for *C. glabrata*. The inhibition was 8.55, 17.88, 35.34, and 77.47% for concentrations 62.5, 125, 250, and 500 μL/L, respectively. The high inhibition activity above 50% was observed for both yeast species at a concentration of 500 μL/L.

To summarize the results, inhibition activity of LCEO in-situ on various types of fruits and vegetables was concentration dependent; as the concentration of LCEO increased, the inhibition of microorganisms was stronger. The strongest inhibition of LCEO was visible on kohlrabi, where 87.70% of *P. megaterium* was inhibited by a concentration of 500 μL/L. The majority of microorganisms (all except *C. tropicalis* on apple and *M. luteus, C. glabrata* on pear) showed strong activity above 50% in a concentration of LCEO of 500 μL/L applied to apple, pear, potato, and kohlrabi. 

Different correlation coefficients were obtained to compare the individual treatments for the analyzed samples for both, antibacterial and antifungal activity. For apple, strong positive correlations were returned for the 250 µL/L and 500 µL/L concentrations for all the bacterial species except *Micrococcus luteus*. For the antifungal activity, the correlation coefficients for this combination of treatments were negative for all the analyzed species. For pear, quite a wide range of correlation coefficients was returned in the analysis, from a nearly absolute negative correlation up to an absolute positive correlation for the individual antibacterial treatment combinations. A similar situation to this occurred in the case of antifungal activity in potato. 

### 2.5. Anti-Insect Activity

The insecticidal activity of LCEO can be considered high (Table 11). The concentration of 6.25% *v/v* of LCEO killed 80% of *Pyrrhocoris apterus.* The 25% *v/v* killed 100% of the individuals. 

## 3. Discussion

Hu et al. [25] found out his LCEO consisted of mainly neral (39.2%) and geranial (30.8%), which together adds up to 70.0% citral. Both isomers of citral reached 68.9%, which is a similar concentration compared to our study. On the other hand, the ratio of isomers was exactly the opposite as in our study where geranial was 39.4% and neral 29.5%. The third major analyzed component was d-limonene at a concentration of 8.28%, which was lower than in our study, where d-limonene was 14.3%. The opposite ratio of isomers compared to our results was shown in Hao et al. [26], where LCEO extracted from fruit was composed of 29.3% of geranial and 38.3% neral. The d-limonene reached 16.5%, which is approximately a 2% higher percentage than the concentration of d-limonene in our study. Yang et al. [22] found out the percentage of geranial was 27.49% and neral was 23.57%. The ratio of isomers was in accordance with our results, but the percentages of both isomers were lower. On the other hand, the percentage of d-limonene was slightly higher (18.82%) than in our study. Chen et al. [27] determined the composition of LCEO distilled from fruit as geranial (37.16%), neral (28.29%), and d-limonene (22.90%), where citral isomers had similar constitution, while d-limonene had a higher percentage compared to our LCEO. Seo et al. [28] determined that LCEO’s main constituents were geranial 39.23%, neral 30.27%, and d-limonene 14.64%, which was the most similar composition to our analyzed LCEO. Si et al. [16] compared the composition of eight LCEOs in their study. The range of geranial content ranged from 44.4% to 50.0% and neral content ranged from 34.2% to 37.4%. Compared to our study, the ratio of citral isomers was similar, but the percentage of components was higher than in our results. On the other hand, the composition of d-limonene was significantly lower than in our study, with the percentage ranging from 0.7 to 5.3%. On the contrary, Hammid and Ahmad [29] found out that LCEO from fruit contained citronellal (51.5%), d-limonene (10.4%), and citronellol (8.9%). Only a small portion of citral (2.6%) was present, which suggests the existence of different LCEO chemotypes other than the citral LCEO chemotype.

Chemical composition is variable, and dependent on the part of the plant [30]. The volume of citral isomers is variable even among different populations [31]. Different seasons of the year and stages of fruit development have an impact on the citral concentration [32]. She et al. [33] suggested that neral concentrations are increased in August which can affect the other biological properties of the LCEO. Moreover, soil composition and fertilization, climatic conditions, and plant cultivation methods can influence the quality and composition of LCEO [34,35]. The prevalent chemotypes of fruit LCEO is citral with d-limonene and citronellal chemotypes [36]. Our LCEO belongs to the citral chemotype, which appeared to be vastly dominant. The characterization of our LCEO correlates with other studies which have evaluated the chemical composition of fruit LCEO.

Thielmann and Muranyi stated [36] that not enough antioxidant activities of LCEO had been evaluated to that date. Currently only a few results can be found which compare the antioxidant activity of LCEO. Pante et al. [37] found out the total antioxidant capacity of LCEO 104.4 mmol Trolox per mg of sample. She et al. [33] found that the IC_50_ values for DPPH radical scavenging were 8.56%, 6.59%, and 4.72%, and varied depending on the month of harvesting. Wang et al. [38] compared antioxidant activity to ascorbic acid and synthetic antioxidants (butylated hydroxytoluene and propyl gallate) and they found out the activity of LCEO was comparable or stronger dependent on the method used for evaluation. Antioxidant activity of LCEO was stronger than that of the citral solution, which suggests the synergism of chemical substances in LCEO. Hwang et al. [39] evaluated the activity of methanol extract, water, butanol, and CHCl_3_ fractions, and the DPPH radical inhibition ranged from 60.25 to 90.57%. These results were approximately two to three times higher than the activity of our tested LCEO, which was 30.9% inhibition and 167.94 TEAC.

Wang and Liu [30] determined inhibition zones of fruit LCEO for G^+^ *Bacillus subtilis, Enterococcus faecalis,* and *Staphylococcus aureus* at 34.5 mm, 18.7 mm, and 30.0 mm, respectively. For G^−^ bacteria *Escherichia coli* and *Pseudomonas aeruginosa* the inhibition zones were 24.1 mm and 18.5 mm, respectively. The inhibition zone for *Candida albicans* was 14.1 mm. The antimicrobial activity was higher for all tested microorganisms compared to our study. Hammid and Ahmad [29] tested inhibition of fruit LCEO against G^+^ bacteria *B. subtilis, S. aureus* where they reached inhibition zones 46.8 mm and 29.0 mm, respectively, while G^−^ bacteria *E. coli, P. aeruginosa* were resistant against LCEO. On the other hand, *S. cerevisiae* yeasts were completely inhibited by fruit LCEO. In our study we reached the opposite activity because the G^−^ bacteria were the most vulnerable to LCEO and yeast strains were the most resistant to tested LCEO. Yang et al. [40] found inhibitory effects only for G^−^ bacteria *Salmonella*, *E. coli* DH5α, *E. coli* O157, and *E. coli* O104 with MIC values 0.05, 0.1, 0.5, and 0.5 μL/mL, respectively. Compared to our study they did not detect any inhibitory activity against G^+^ bacteria. The minimum inhibitory concentration against methicillin resistant *S. aureus* was 0.5 mg/mL, which was considered high antimicrobial activity by the authors Hu et al. [25]. Compared to our study, the antimicrobial activity against G^+^ bacteria was considered moderate to strong. Hao et al. [26] found out that LCEO significantly inhibited to the growth of G^−^ bacteria *Acinetobacter baumannii,* and they determined its MIC value at 1.04 mg/mL. In our study, the inhibition of G^−^ bacteria was strong, which correlates with recent findings.

Saikia et al. [41] observed that the differences in antimicrobial activity were connected with main components of LCEO (from India). The LCEO which contained nerol and geraniol as main components showed inhibitory activity against *S. aureus* with inhibition zone 15 mm and MIC 1.25 mg/mL, *L. monocytogenes* 14 mm with MIC 2.5 mg/mL, *E. coli* 21 mm and MIC 10 mg/mL, and *P. aeruginosa* 8 mm and MIC 10 mg/mL. The activity of LCEO against *C. albicans* was strong where diluted LCEO reached the inhibition zone by over 30 mm with MIC 2.5 mg/mL. The author stated that LCEO of the citral chemotype was the most active against microorganisms. Conversely, it has the best inhibition activity against G^+^ bacteria and yeasts.

The antimicrobial activity of LCEO on fresh fruits or vegetables has not been tested so far. In our laboratory, previous analyses suggested that essential oils from lemongrass, wild thyme, and cinnamon were effective against potential pathogens on potato and carrot in the vapor phase [42,43,44]. Tyagi and Malik [45] found out that vapor phase of Lemongrass essential oil with major constituents d-limonene and citral was effective against *E. coli* strains. Fancello et al. [46] tested the gaseous phase of *Citrus limon* EO on ricotta cheese. The essential oil prepared from leaves with the presence of d-limonene and citral in the gaseous phase showed inhibition activity against *L. monocytogenes*. Fancello et al. [47] also found out that citral from *Citrus limon* EO contributes to antimicrobial activity in the gaseous phase.

The anti-insect activity of our LCEO was strong against *P. apterus,* where 25% *v/v* killed 100% of individuals. Yang et al. [22] found out that fumigant toxicity of essential oil of LCEO against *Lasioderma serricorne* was high with an LC_50_ value of 22.97 mg/L air. Against *Liposcelis bostrychophila,* the LC_50_ value was determined to be 0.73 mg/L air. They also found out that citral greatly contributes to the toxicity of LCEO against selected insects. The LCEO from a mature fruit grown in Thailand showed significant fumigant toxicity against cereal pests *Sitophilus zeamais* and *Tribolium castaneum,* with LC_50_ values 92.46 µL/L and 549.57 µL/L, respectively [23]. LCEO characterized by Jiang et al. [48] observed moderate toxic activity against *Trichoplusia ni* larvae with LD_50_ 112.5 μg/larva. Seo et al. [28] detected that LCEO from Vietnam was also highly active against *Reticulitermes speratus,* where 78% of individuals were dead at a concentration of 1.5 mg/filter paper and 100% of individuals were dead at a concentration of 2 mg/filter paper after two days. They also observed that constituents citral and d-limonene, which were present in LCEO, contributed to the anti-insect activity. Wang et al. [49] tested the activity of LCEO against *Tenebrio molitor* larvae and found 75.6% mortality after 48 h and 92.2% mortality after 96 h. Antiinsect activity of EO gainst *Alphitobius diaperinus* adults showed 12.2% of dead individuals, which were found after 48 h and 23.3% of dead individuals, which were found after 96 h [50].

## 4. Materials and Methods

LCEO was purchased from the Hanus company. The provider stated that commercial essential oil was prepared by distillation of *L. cubeba* fruits from China. 

### 4.1. Microorganisms

The G^+^ bacteria (*Micrococcus luteus* CCM 732, *Bacillus* since 2020 *Priestia megaterium* CCM 2007), G^−^ bacteria (*Azotobacter chroococcum* CCM 1912, *Serratia marcescens* CCM 8587), and yeasts (*Candida glabrata* CCM 8270, *Candida tropicalis* CCM 8264), obtained from Czech Collection of Microorganisms (Brno, Czech Republic), were used for analyses of antimicrobial activity. 

### 4.2. Gas Chromatography Analysis Conditions

Chemical composition of LCEO was determined with gas chromatography mass spectrophotometry (GC-MS) and gas chromatography/flame ionization detector (GC-FID) analyses. The individual volatile constituents of EO were identified according to retention indices on capillary column HP-5MS [51]. A capillary column HP-5MS (30 m × 0.25 mm × 0.25 μm) was used. The temperature program was set from 60 °C to 150 °C (increasing rate 3 °C/min) and 150 °C to 280 °C (increasing rate 5 °C/min) with a total run time of 60 min. The carrier gas was helium 5.0 with a flow rate of 1 mL/min. The split/splitless injector temperature was set at 280 °C and 1 μL of LCEO sample diluted in pentane (10% solution) was injected. The sample was injected in the split mode with a split ratio of 40.8:1. Electron-impact mass spectrometric data (EI-MS; 70 eV) were acquired in scan mode over the m/z range 35–550. The temperature of the MS ion source was 230 °C and the MS quadrupole temperature was 150 °C. GC-FID analyses were performed on Agilent 6890N coupled to an FID detector. Column and chromatographic conditions were the same as in GC-MS analysis. The temperature of the FID detector was set at 300 °C. The volatile constituents of EO were identified according to retention indices and were compared to reference spectra (Wiley and NIST databases). The retention indices were experimentally determined using the standard method of n-alkanes (C6-C34) retention times, and injected under the same chromatographic conditions [52]. The percentages of the compounds with concentrations higher than 0.1% were derived from GC peak areas. The percent area for each compound was calculated without using the response factors. The analyses were performed in triplicate and the average value was calculated.

### 4.3. Antioxidant Activity

The antioxidant activity of LCEO was determined by the scavenging of the DPPH (Sigma Aldrich, Schnelldorf, Germany) radical. A methanolic solution of DPPH (0.025 g/L) was adjusted to an absorbance of 0.8 at 515 nm (Glomax spectrophotometer, Promega Inc., Madison, WI, USA); 5 μL of the EO sample was added to 195 μL DPPH and was incubated for 30 min in the dark during continual shaking at 1000 rpm. The inhibition activity was calculated as (A0-AA)/A0 × 100, where A0 was absorbance of control (195 μL DPPH + 5 μL methanol) and AA was absorbance of sample. 

The total antioxidant capacity (TEAC) was calculated according to the standard reference Trolox (Sigma Aldrich, Schnelldorf, Germany), as described in [43]. 

### 4.4. Antimicrobial Activity

The Kirby-Bauer disc diffusion method was used for LCEO antimicrobial activity determination described in [53]. Microbial inoculum was cultivated for 24 h on Tryptone soya agar (TSA, Oxoid, Basingstoke, UK) at 37 °C (bacteria) or Sabouraud dextrose agar (SDA, Oxoid, Basingstoke, UK) at 25 °C (yeasts). The microbial culture was adjusted to a density of 0.5 McFarland standard (1.5 × 10^8^ CFU/mL) and 100 μL of the microbial culture was prepared on Mueller Hinton agar (MHA, Oxoid, Basingstoke, UK) for bacteria and on SDA for yeasts; 6 mm discs were placed on microbial culture and 10 μL of LCEO were added to the discs. Samples were incubated at 37 °C (bacteria) or 25 °C (yeasts) for 24 h. The inhibition zones were measured three times from the edge of the filter. The inhibition activity was classified as: larger than 10 mm = very strong antimicrobial activity; 10–5 mm = moderate activity; 5–1 mm = weak activity. The antibiotics were used as control: cefoxitin and gentamicin for bacteria, fluconazole for yeasts. 

MIC was evaluated by broth microdilution method in a 96 well plate according to [53]. Microbial inoculum was cultivated for 24 h in Mueller Hinton Broth (MHB, Oxoid, Basingstoke, UK) at 37 °C (bacteria) or Sabouraud dextrose broth (SDA, Oxoid, Basingstoke, UK) at 25 °C (yeasts); 50 μL of microbial suspension was adjusted to optical density 0.5 McFarland and was added to a 96-well microplate. Next, 100 μL of LCEO was added to microbial suspension diluted to final concentrations ranging from 400 μL/mL to 0.2 μL/mL. Samples were mixed and incubated for 24 h at 25 °C (yeast cultures) or 37 °C (bacterial cultures). MHB with EO was used as a negative control and MHB with inoculum was used as a positive control. Absorbance was measured spectrophotometrically at the beginning of the experiment and after 24 h at 570 nm. Both analyses were prepared in triplicate. 

### 4.5. Antimicrobial Activity In-Situ on Food Models

The in-situ antimicrobial analyses were performed on two types of fruit (apple and pear) and two types of vegetable (potato and kohlrabi) as previously described in [54]. The percentage of inhibitory activity was calculated in ImageJ by stereological method. Bulk density was calculated according to the formula Vv = P/p × 100 where P is stereological lattice of the colonies and p is the substrate. Growth inhibition was expressed as GI = [(C−T)/C] × 100, where C was the growth density of control group and T was the growth density in the group contained LCEO.

### 4.6. Anti-Insect Activity

The activity of LCEO against insects was tested on *Pyrrhocoris apterus*. The LCEO was used in concentrations 100%, 50%, 25%, 12.5%, and 6.25%. Live and dead specimens were counted and percentage of inhibition was calculated [54]. 

### 4.7. Statistical Analysis

One-way analysis of variance (ANOVA) was performed using Prism 8.0.1 (GraphPad Software, San Diego, CA, USA) followed by Tukey’s test at *p* < 0.05. SAS^®^ software version 8 was used for data processing. F-test and correlation coefficients were calculated in MS Excel and Data analysis utility.

The MIC concentration values of 50% and 90% growth inhibition were calculated by probit analysis. All analyses were performed in three replications. 

## 5. Conclusions

The antioxidant and antimicrobial activity of LCEO was evaluated related to its potential as a microbial and insect inhibitor. The main components geranial, neral, and d-limonene were analyzed. The LCEO showed moderate antioxidant activity. The disk diffusion method showed strong antimicrobial activity against *A. chroococcum*, *S. marcescens,* and *P. megaterium*. The minimum inhibitory concentration showed the best effectivity of LCEO against *M. luteus.* The vapor phase of LCEO was effective against all tested microorganisms at a concentration of 500 µL/mL and the activity was concentration dependent. The vapor phase of LCEO was also very effective against the insect *P. apterus.* The LCEO showed very good antimicrobial properties that could be applied to inhibit food spoilage and extension of the shelf life of the food products. LCEO showed good potential as an anti-insect agent, which could also be tested on food stuff with pests that deteriorate the quality of foods. Research could be extended to food products with an emphasis on sensory properties.

According to the Food and Drug administration, components of LCEO are generally considered safe. The European Food Safety Authority stated that the main components of LCEO and whole EO are safe and do not possess genotoxic or cancerogenic properties. The maximum proposed level is safe in the range 8.5–125 mg/kg for animals as feed additive. The components are registered as flavorings and can be used as food supplements. Thus, LCEO can be examined as a potential antimicrobial and antioxidant agent in food products and food resources.

## Figures and Tables

**Table 1:** Chemical composition of LCEO.

Experimental RI ^a^	Literature RI	Identified Compounds	Content [%] ^b^
1266	1267	geranial	39.4 ± 0.67
1238	1238	neral	29.5 ± 0.54
1028	1029	limonene	14.3 ± 0.26
980	979	β-pinene	2.3 ± 0.11
977	975	sabinene	1.9 ± 0.09
1033	1031	1,8-cineole	1.9 ± 0.08
938	939	α-pinene	1.7 ± 0.13
1088	1088	α-terpinolene	1.6 ± 0.11
983	985	6-methyl-5-hepten-2-one	1.3 ± 0.07
1178	1177	4-terpinenol	0.9 ± 0.03
1256	1252	geraniol	0.9 ± 0.02
1422	1419	(*E*)-caryophyllene	0.9 ± 0.05
1189	1188	α-terpineol	0.8 ± 0.03
1152	1153	citronellal	0.7 ± 0.02
1583	1583	caryophyllene oxide	0.5 ± 0.01
948	954	camphene	0.4 ± 0.02
1227	1229	nerol	0.4 ± 0.02
total			99.3 ± 0.18

^a,^ Retention indices on HP-5MS column; ^b,^ medium value of percentage area of three independent injections.

**Table 2:** Antimicrobial activity of LCEO determined by disk diffusion and broth microdilution methods.

Microorganisms	Inhibition zone (mm)	Activity of EO	MIC 50 (µL/mL)	MIC 90 (µL/mL)	Antibiotics (mm)
Gram-negative bacteria					
*Azotobacter chroococcum*	11.33 ± 0.58	***	6.35	8.42	27.33 ± 1.15
*Serratia marcescens*	14.33 ± 0.58	***	2.36	4.28	29.33 ± 0.58
Gram-positive bacteria					
*Priestia megaterium*	11.33 ± 0.58	***	3.46	26.54	31.33 ± 0.58
*Micrococcus luteus*	9.67 ± 0.58	**	1.46	3.52	27.33 ± 0.58
Yeasts					
*Candida glabrata*	5.33 ± 0.58	**	6.18	7.45	29.33 ± 0.58
*Candida tropicalis*	7.33 ± 0.58	**	6.24	8.36	30.33 ± 0.58

Weak (0–5 mm, *), moderate (5–8 mm, **), and strong (>8 mm, ***) antimicrobial activity.

**Table 3:** Inhibition activity of LCEO against bacteria on an apple.

	Growth Inhibition [%]			
*Litsea Cubeba* EO (µL/L)	*Azotobacter Chroococcum*	*Priestia Megaterium*	*Serratia Marcescens*	*Micrococcus Luteus*
62.5	26.63 ± 4.53 ^a^	14.72 ± 1.95 ^a^	13.68 ± 3.19 ^a^	6.39 ± 1.01 ^a^
125	33.23 ± 6.37 ^a^	32.99 ± 1.98 ^b^	25.41 ± 4.51 ^a^	13.38 ± 0.95 ^b^
250	48.71 ± 2.47 ^b^	46.12 ± 4.05 ^c^	35.72 ± 3.41 ^b^	28.22 ± 3.00 ^c^
500	58.31 ± 4.47 ^b^	74.69 ± 3.99 ^d^	76.16 ± 4.11 ^c^	75.82 ± 3.64 ^d^
R^2^	0.9789	0.9750	0.8843	0.8488

^a, b, c, d^ indicate significant differences between concentrations of the same column and for each treatment with *p* ≤ 0.05.; three replicates of each treatment were performed; Anova test was used as statistical test.

**Table 4:** Inhibition activity of LCEO against yeasts on an apple.

	Growth Inhibition [%]	
*Litsea Cubeba* EO (µL/L)	*Candida Glabrata*	*Candida Tropicalis*
62.5	4.38 ± 1.05 ^a^	4.51 ± 2.07 ^a^
125	11.34 ± 1.63 ^a^	7.03 ± 1.47 ^a^
250	26.42 ± 1.76 ^b^	14.00 ± 2.33 ^b^
500	53.91 ± 4.11 ^c^	26.31 ± 3.33 ^c^
R^2^	0.9265	0.9160

^a, b, c,^ indicate significant differences between concentrations of the same column and for each treatment with *p* ≤ 0.05.; three replicates of each treatment were performed; Anova test was used as statistical test. The inhibition of bacteria and yeasts by LCEO on apple was concentration dependent for every microorganism. The higher concentration of LCEO showed stronger activity against selected microorganisms. A statistical difference of each concentration was visible in *P. megaterium* and *M. luteus*.

**Table 5:** Inhibition activity of LCEO against bacteria on a pear.

	Growth Inhibition [%]			
*Litsea Cubeba* EO (µL/L)	*Azotobacter Chroococcum*	*Priestia Megaterium*	*Serratia Marcescens*	*Micrococcus Luteus*
62.5	7.15 ± 1.79 ^a^	5.94 ± 1.72 ^a^	5.89 ± 0.61 ^a^	5.41 ± 0.74 ^a^
125	17.49 ± 2.37 ^b^	14.32 ± 0.89 ^b^	25.41 ± 4.51 ^a^	16.10 ± 3.31 ^b^
250	45.79 ± 3.96 ^c^	29.84 ± 3.44 ^c^	35.72 ± 3.41 ^b^	22.94 ± 2.54 ^b^
500	66.79 ± 4.16 ^d^	76.01 ± 2.96 ^d^	76.16 ± 4.11 ^c^	45.51 ± 3.27 ^c^
R^2^	0.9727	0.8688	0.9290	0.9369

^a, b, c, d^ indicate significant differences between concentrations of the same column and for each treatment with *p* ≤ 0.05.; three replicates of each treatment were performed; Anova test was used as statistical test.

**Table 6:** Inhibition activity of LCEO against yeasts on a pear.

	Growth Inhibition [%]	
*Litsea Cubeba* EO (µL/L)	*Candida Glabrata*	*Candida Tropicalis*
62.5	2.46 ± 0.95 ^a^	12.62 ± 2.50 ^a^
125	4.75 ± 1.25 ^a^	26.69 ± 5.66 ^b^
250	10.14 ± 2.11 ^b^	46.59 ± 3.01 ^c^
500	22.47 ± 2.66 ^c^	87.02 ± 3.68 ^d^
R^2^	0.8919	0.9412

^a, b, c, d^ indicate significant differences between concentrations of the same column and for each treatment with *p* ≤ 0.05.; three replicates of each treatment were performed; Anova test was used as statistical test. The inhibition of microorganisms grown on a pear by LCEO was concentration dependent and inhibition of microorganisms was higher with increasing concentration. The statistical differences between each concentration were visible in bacteria *A. chroococcum*, *P. megaterium* and yeast *C. tropicalis*.

**Table 7:** Inhibition activity of LCEO against bacteria on a potato.

	Growth Inhibition [%]			
*Litsea Cubeba* EO (µL/L)	*Azotobacter Chroococcum*	*Priestia Megaterium*	*Serratia Marcescens*	*Micrococcus Luteus*
62.5	6.02 ± 1.88 ^a^	3.92 ± 1.53 ^a^	7.34 ± 2.60 ^a^	12.68 ± 2.61 ^a^
125	12.42 ± 1.11 ^b^	12.76 ± 1.65 ^b^	16.05 ± 3.93 ^a^	27.64 ± 4.69 ^b^
250	33.32 ± 1.89 ^c^	23.88 ± 1.62 ^c^	24.35 ± 2.79 ^b^	42.35 ± 2.85 ^c^
500	56.68 ± 1.60 ^d^	62.13 ± 2.14 ^d^	76.35 ± 3.05 ^c^	85.32 ± 3.83 ^c^
R^2^	0.9493	0.9647	0.8937	0.9436

^a, b, c, d^ indicate significant differences between concentrations of the same column and for each treatment with *p* ≤ 0.05.; three replicates of each treatment were performed; Anova test was used as statistical test.

**Table 8:** Inhibition activity of LCEO against yeasts on a potato.

	Growth Inhibition [%]	
*Litsea Cubeba* EO (µL/L)	*Candida Glabrata*	*Candida Tropicalis*
62.5	6.50 ± 1.80 ^a^	6.41 ± 1.79 ^a^
125	14.38 ± 3.84 ^a^	15.78 ± 2.32 ^b^
250	33.99 ± 4.66 ^b^	33.69 ± 2.12 ^c^
500	63.23 ± 2.94 ^c^	68.45 ± 3.13 ^d^
R^2^	0.9403	0.9267

^a, b, c, d^ indicate significant differences between concentrations of the same column and for each treatment with *p* ≤ 0.05.; three replicates of each treatment were performed; Anova test was used as statistical test. The activity of LCEO was also concentration dependent against microorganisms that were growing on potato. The inhibition of microorganisms was stronger with increasing concentration. The statistical differences between each concentration were observed in *A. chroococcum*, *P. megaterium,* and *C. tropicalis*.

**Table 9:** Inhibition activity of LCEO against bacteria on a kohlrabi.

	Growth Inhibition [%]			
*Litsea Cubeba* EO (µL/L)	*Azotobacter Chroococcum*	*Priestia Megaterium*	*Serratia Marcescens*	*Micrococcus Luteus*
62.5	6.24 ± 1.02 ^a^	5.47 ± 1.83 ^a^	7.64 ± 1.45 ^a^	15.24 ± 3.21 ^a^
125	17.60 ± 2.38 ^b^	23.32 ± 2.57 ^b^	12.62 ± 2.84 ^b^	25.35 ± 2.94 ^b^
250	46.83 ± 3.28 ^c^	47.20 ± 3.85 ^c^	35.81 ± 4.06 ^c^	44.94 ± 3.66 ^c^
500	85.55 ± 3.22 ^d^	87.70 ± 5.10 ^d^	75.99 ± 4.99 ^d^	77.59 ± 1.25 ^d^
R^2^	0.9497	0.8747	0.8038	0.9195

^a, b, c, d^ indicate significant differences between concentrations of the same column and for each treatment with *p* ≤ 0.05.; three replicates of each treatment were performed; Anova test was used as statistical test.

**Table 10:** Inhibition activity of LCEO against yeasts on a kohlrabi.

	Growth Inhibition [%]	
*Litsea Cubeba* EO (µL/L)	*Candida Glabrata*	*Candida Tropicalis*
62.5	8.55 ± 2.33 ^a^	7.33 ± 1.26 ^a^
125	17.88 ± 1.91 ^b^	14.68 ± 2.07 ^b^
250	35.34 ± 3.34 ^c^	30.94 ± 2.36 ^c^
500	77.47 ± 2.05 ^d^	66.70 ± 3.69 ^d^
R^2^	0.8989	0.9011

^a, b, c, d^ indicate significant differences between concentrations of the same column and for each treatment with *p* ≤ 0.05.; three replicates of each treatment were performed; Anova test was used as statistical test. Growth of the microorganisms by LCEO was also inhibited on kohlrabi. With growing concentration of LCEO the inhibition was stronger against all microorganisms. Statistical differences of every concentration was observed at all four bacteria and both tested yeasts.

**Table 11:** Anti-insect activity of LCEO against *P. apterus*.

Concentration [%]	Number of Living Individuals	Number of Dead Individuals	Insecticidal Activity [%]
100	0	30	100
50	0	30	100
25	0	30	100
12.5	3	27	90
6.25	6	24	80
Control group	30	0	0

## Data Availability

Not applicable.
